# Skin Models Used to Define Mechanisms of Action of Sulfur Mustard

**DOI:** 10.1017/dmp.2023.177

**Published:** 2023-10-18

**Authors:** Jeffrey D. Laskin, Kevin Ozkuyumcu, Peihong Zhou, Claire R. Croutch, Diane E. Heck, Debra L. Laskin, Laurie B. Joseph

**Affiliations:** 1 Department of Environmental and Occupational Health and Justice, Rutgers University School of Public Health, Piscataway, NJ, USA;; 2 Department of Pharmacology and Toxicology, Ernest Mario School of Pharmacy, Piscataway, NJ, USA; 3 MRIGlobal, Kansas City, MO, USA

**Keywords:** animal models, dermatotoxicity, sulfur mustard, wound healing

## Abstract

Sulfur mustard (SM) is a threat to both civilian and military populations. Human skin is highly sensitive to SM, causing delayed erythema, edema, and inflammatory cell infiltration, followed by the appearance of large fluid-filled blisters. Skin wound repair is prolonged following blistering, which can result in impaired barrier function. Key to understanding the action of SM in the skin is the development of animal models that have a pathophysiology comparable to humans such that quantitative assessments of therapeutic drugs efficacy can be assessed. Two animal models, hairless guinea pigs and swine, are preferred to evaluate dermal products because their skin is morphologically similar to human skin. In these animal models, SM induces degradation of epidermal and dermal tissues but does not induce overt blistering, only microblistering. Mechanisms of wound healing are distinct in these animal models. Whereas a guinea pig heals by contraction, swine skin, like humans, heals by re-epithelialization. Mice, rats, and rabbits are also used for SM mechanistic studies. However, healing is also mediated by contraction; moreover, only microblistering is observed. Improvements in animal models are essential for the development of therapeutics to mitigate toxicity resulting from cutaneous exposure to SM.

Sulfur mustard (SM, bis 2-chloroethyl sulfide) is a potent skin vesicant synthesized for chemical warfare. As a bifunctional alkylating agent, SM initiates its action by modifying and disrupting cellular macromolecules, including DNA and proteins.^1–5^ Acute responses of skin to SM are typically characterized by delayed onset erythema and intense itching, followed by the formation of small fluid-filled vesicles; with time, these vesicles coalesce to form pendulous blisters.^1,6,7^ A necrotic layer and ulceration can form on the affected skin surface following rupture of the blisters. Responses of human skin to SM are multifactorial and depend on the dose and time following exposure, as well as environmental conditions such as temperature and humidity.^7,8^ Location of exposure sites on the body, variations in skin properties, and underlying disease states, along with age and sex, are all determinants of skin responses to SM.^8^

To understand the mechanism of action of SM and develop medical countermeasure, various animal models have been utilized, including mice, rats, guinea pigs, rabbits, and pigs.^9–12^ Unfortunately, there are no simple or common animal models for SM injury that produce true blisters like humans. In this context, in describing early reporting on the use of human subjects for mustard research in 1919, Sollman explained that “experiments on animals was *[sic]* abandoned after a few trials, since their skin does not react in the same manner as human skin, and the effects that do occur are not easily graded.”^8^ Blistering is not commonly observed in animals.^13,14^ To produce true blistering, either unconventional species must be used, or multistep procedures must be undertaken in common animal models.^14^ For example, it has been reported that blisters can be produced on the skin of frogs, birds, and the inner ears of rabbits,^15^ on the skin of isolated perfused pig flaps,^15,16^ and on guinea pig skin that has been thermally burned and allowed to re-epithelialize.^16,17^ Studies performed with SM on birds and frogs are limiting as their skin is not similar to human skin. For this reason, SM research has relied on the surrogate marker of microblistering or subepidermal blister formation at the dermal-epidermal junction, which occurs in rodents, rabbits, and pigs.^18–20^ SM is known to damage not only epidermal structures, including the basement membrane, but also stromal and vascular components of the skin tissue.^21–23^

Translating SM data from animals to humans has been challenging not only because there is little or no blistering, but also due to additional factors such as distinct structural differences in the tissue, unique aspects of the immune system, and mechanisms of wound healing. For example, in mice, the skin and epidermis are thinner when compared to humans, there are fewer epidermal cell layers, a lack of epidermal ridges and eccrine sweat glands, and limited adherence to underlying tissues.^24^ In humans and pigs, wounds close by formation of granulation tissue followed by re-epithelialization^24^; in contrast, wound closure in rodents and rabbits is primarily by contraction, in part due to the presence of the panniculus carnosus.^25^ At later stages, tissue remodeling during wound healing occurs via fibroblast migration and myofibroblast activity.^26^ It should be noted that contraction is usually defined for incisional wounds^27^; the role of contraction in thermal and SM injury is not clear since the extent of tissue damage may not allow wound closure by the panniculus carnosus.

In rodent models, both haired and hairless strains have been used; hairless animals are advantageous largely due to the ease of visualizing a cutaneous response.^28^ Hair removal and associated inflammation are avoided with these animals.^29^ However, it should be noted that the skin of haired and hairless animal strains can be morphologically different. For example, the epidermis of mice of the most commonly used hairless strain, SKH1, is thicker than haired strains.^9^ Haired and hairless strains are also genetically and immunologically distinct, complicating efforts to compare results from different laboratories.^30^ Little information is available on differences in wound healing in response to chemical and thermal injury in haired and hairless mouse strains.

In most animal models, different phases of SM injury can be defined, including latency, erythema/inflammation, microblistering, ulceration/eschar formation, and wound healing. The extent of injury depends on several factors, including the model, location and area of skin exposed to SM, as well as SM dose and methods of administration and environmental conditions when applying SM. Targeting one or more phases of injury is essential in the development of effective countermeasures to mitigate SM toxicity. Both clinical signs and morphological/biochemical parameters have been used to characterize the action of SM in animal models. Clinical signs are evident by visual inspection; at early times, this includes erythema, edema and transepidermal water loss (TEWL), and, at later times, extent of injury and whether injury is superficial, intermediate in depth, or deep dermal injury.^29^ The integrity of the dermal-epidermal junction, measured by dermal torque, has been demonstrated in SM-treated guinea pigs.^31^ Laser Doppler imaging has also been used to assess cutaneous blood flow and ballistometry to evaluate mechanical properties of the skin, including rigidity and elasticity in pig models.^32,33^

Techniques in histology, electron microscopy, and immunohistochemistry have been used to analyze structural alterations in skin exposed to SM. These studies have largely focused on the epidermis, basement membrane, and accessary structures, including hair follicles and sebaceous glands. Early effects of SM in basal cells of the epidermis, as reported in guinea pig skin include nuclear condensation and mitochondrial swelling, disorganization of desmosomes and hemidesmosomes, and widening of intracellular spaces in the basal cell layer.^19^ At later times, nuclear pyknosis, cell fragmentation, and necrosis extending into suprabasal cells are evident.^34^ Markers of DNA damage and apoptosis and necrosis also appear in epidermal cells.^35^ Mediators of inflammation, including prostaglandins and cytokines, are also expressed after SM-induced injury.^11,22^ Microvesicles appear in the lamina lucida of the basement membrane as a consequence of degeneration of the basal layer.^36,37^ Proteolysis of basement membrane components, including laminins, collagens, and other anchoring proteins by matrix metalloproteinases, contributes to the disruption of the basal cell layer, microvesication, and ulceration.^38,39^ At later times, in minipig skin, aberrant epidermal proliferation and differentiation are associated with re-epithelialization including hyperplasia, hyperkeratosis, and parakeratosis. This is thought to contribute to prolonged wound healing.^40,41^

The dermis and hypodermis are also targets for SM. This is important as the integrity of these tissues is critical for wound healing.^22,23,42,43^ Leukocyte infiltration, a marker of inflammation, has been observed in the dermis post-SM exposure in all animals studied.^19,44–46^ In mouse and guinea pig skin, mast cell degranulation is also evident, along with alterations in collagen deposition.^38,47,48^ In pig skin, SM also disrupts the dermal vasculature and subsequent blood flow, and responses can affect tissue oxygenation, possibly leading to reperfusion injury.^49,50^ These pathologic responses can impair wound healing, lead to infection, and initiate scarring.

## Guinea Pig Skin Model of SM Toxicity

Both haired and hairless guinea pigs have been used to assess SM toxicity with generally similar results (Table 1). Hairless guinea pigs have been reported to be more sensitive to SM in terms of the extent of dermal injury.^51^ These animals are also more sensitive to SM-induced epidermal necrosis compared to other animal models, including the weanling pig, mouse ear, and hairless mice.^18^ The hairless guinea pig skin is considered morphologically more like human skin,^31,52^ which has prompted greater use of these animals to understand the mechanism of action of SM and for the development of countermeasures.^53^

As indicated above, a characteristic early response of guinea pig skin to SM is a marked inflammatory response, notably, infiltration of neutrophils and macrophages into the tissue.^54^ Mustards cause the release of inflammatory mediators, including reactive oxygen and reactive nitrogen species, and cytokines such as TNFα and IL-1α, which activate macrophages contributing to tissue injury.^2,55^ This is followed by the appearance of anti-inflammatory/wound repair macrophages.^56^ That macrophages can contribute to wound repair is evidenced by findings that intradermal injection of activated human macrophages into SM-treated guinea pig skin can significantly improve clinical signs of tissue damage.^57^

Of interest are studies by Graham et al.^47^ showing that SM reduces mast cell numbers in hairless guinea pig skin, suggesting that degranulation may be an early marker of toxicity. These investigators hypothesized that histamine and other mediators released by mast cells may play a role in SM-induced injury. These data are in accord with studies by ours and other laboratories demonstrating mast cell degranulation and reduced number of mast cells in SM-exposed hairless mouse skin.^38,48^ The use of antihistamine promethazine, in combination with the PARP inhibitor niacinamide, and the non-steroidal anti-inflammatory agent indomethacin in guinea pig skin, decreases mast cell degranulation.^58–60^

## Rat, Mouse, and Rabbit Models of SM Toxicity

In these models, exposure to SM is either by direct application of liquid to the skin or as a vapor (Tables 2–5). Vapor exposures are typically preferred since vapor is the more likely route of exposure during a mass causality scenario. Depending on the dose and environmental conditions, generally similar characteristic responses are observed following treatment of the dorsal skin of rats, mice, and rabbits with SM. Initially, there is a latency period, which is followed by a cutaneous inflammatory response characterized by erythema, edema, and leukocyte infiltration. Subsequently, there is microblister formation, tissue granulation, epidermal necrosis, and, finally, wound repair and tissue remodeling.^15,61,62^ More detailed information has been reported on the effects of SM on hair follicles and sebaceous glands in the mouse model.^23,63^ In hair follicles, SM induces epithelial cell karyolysis within the hair root sheath, infundibulum, and isthmus and reduces the numbers of sebocytes in sebaceous glands.^64^ Significant DNA damage and apoptosis are evident around pilosebaceous units with increased numbers of inflammatory cells surrounding utriculi. These findings may explain, at least in part, depletion of hair follicles in human skin following exposure to SM.

An important method that can partially overcome wound contraction and the need for fur removal is the use of the mouse ear vesicant model (see Table 4). This method is largely based on early studies showing that biological and biochemical processes associated with inflammation can easily be measured following exposure to cutaneous irritants or allergens.^36,65–67^ In this model, SM is applied to the inner surface of the mouse ear, which is largely free of hair. Ear cartilage appears to prevent wound contraction.^68^ After a latency period, edema, measured by changes in ear weight, epidermal necrosis, and epidermal-dermal separation are assessed.^65,69^ Transmission electron microscopy and immunohistochemistry have been used to identify biomarkers of injury, as well as mechanisms of subepidermal blister formation.^36,70^

In rabbits, dorsal and ventral skin and ear skin have been used to investigate SM injury and the formation of microblisters.^71–75^ In each exposure scenario, SM damage has been assessed visually by monitoring erythema, wound healing, and histopathology.^19,73,75,76^ In the rabbit models, depending on the dose, SM damages the superficial microvasculature as measured by Evans blue dye extravasation and leakage of erythrocytes.^19,77^ SM also damages fibroblasts, possibly disrupting the extracellular matrix. In contrast to the dorsal and ventral skin, rabbit ears have no panniculus carnosus; thus, wound contraction does not contribute to the healing process.^78^ This model is thought to better reflect wound healing in humans. However, in a continuous flow vapor exposure model, rabbit ears have been reported to be significantly less sensitive than human skin to SM injury.^76^

## Pig Models of SM Toxicity

Pig skin is the most anatomically and physiologically similar to human skin, compared to rodents and rabbits, making it a preferable model for translational research (Tables 6 and 7). From a regulatory standpoint, considerable background data are available on pig skin related to the development of dermatological products, making this model ideal for SM countermeasure research. Pig skin is tightly attached to the subcutaneous connective tissue, contains a relatively thick epidermis, distinct rete ridges and, like human skin, dense elastic fibers in the dermis.^79–81^ Pig skin hair is coarser than human hair but has a similar distribution.^41,79,82^ Although humans have eccrine glands distributed throughout their skin, swine eccrine glands are primarily found in the snout, lips, and carpal organ.^80^ In the skin of both pigs and humans, re-epithelialization during wound healing is associated with basal cell proliferation and differentiation into enucleated granular cells that migrate outward toward the surface of the skin.^83^ However, as with other animal models, SM is unable to form true blisters, a characteristic sign of toxicity in humans following vesicant exposure.^6,7,84^

Both dorsal and ventral skin models have been used to assess SM toxicity in pig skin (see Tables 6 and 7). In general, the ventral skin of pigs is thinner and more responsive to SM than dorsal skin.^85^ The choice of dorsal versus ventral pig skin models is dependent on the type of exposure (eg, liquid vs vapor cap) and the type of injury being investigated (eg, superficial vs intermediate or deep dermal). Both models can be used to assess pharmaceutical preparations. However, dorsal skin is preferable with the use of wound dressings that must be maintained for prolonged periods of time (see further below). Both clinical and histopathological endpoints are used to assess tissue damage. Clinical changes include blood flow, elasticity, skin color, thickness, and spectral properties.^49,50^ Histopathological changes include skin structure, epithelial and basement membrane integrity, and expression of markers of proliferation and differentiation of keratinocytes during wound healing.^40,86–88^ Following these endpoints over time will provide information on the wound healing process and the effectiveness of potential countermeasures. Decontaminants, protectants, anti-inflammatory agents, and wound dressing have been evaluated for their ability to mitigate tissue damage induced by SM, often with varying degrees of success.^89^

Based on pig skin models that have been developed to assess medical countermeasures against SM-induced skin injury, one product, Silverlon^®^ Wound Contact, Burn Contact Dressings, has been approved by the FDA.^90^ Manufactured as a non-adherent knitted nylon fiber wound dressing coated with metallic silver, Silverlon^®^ is approved for use with decontaminated, unroofed first and second degree burns induced by SM. Silverlon^®^ also acts as an oxygen-permeable sterile barrier, which promotes wound healing.^53^ Silver ions in the product also serve as an antimicrobial, reducing infections at the wound site.^91^

Support for Silverlon^®^ in the FDA approval process was based on a pathophysiological scale in the Göttingen minipig vapor cap model (Table 8). Individual endpoints indicate the extent and type of repair and include the appearance of epithelial cells, basement membrane damage, re-epithelialization of the wound, whether abnormal hair follicles are present, extent of dermal inflammation and the presence of rete ridges, vascular proliferation, and hemorrhage.^33,92^ In the case of Silverlon^®^, approvals were based on re-epithelialization of the skin and improved appearance of the basement membrane, as well as a reduction in dermal inflammation. Silverlon^®^ has also been FDA approved for radiation dermatitis and cutaneous radiation injury through dry desquamation.^93^

## Summary

Animal models are essential not only for understanding the mechanism of action of SM, but also to develop effective therapeutics. Importantly, therapeutics may be effective at different stages of SM injury (eg, during the latency prior to a cutaneous response, during the inflammatory response, or during wound healing/tissue remodeling) and can be used alone or in combination. For example, Silverlon^®^ is effective for wound healing following the appearance of first- and second-degree burns after exposure to SM. It remains to be determined whether treatments with anti-inflammatory agents prior to the development of SM burns will improve Silverlon^®^-induced wound healing. Thus far, research in the field is limited as SM is a blistering agent, and none of the animal models form overt blisters in response to this vesicant. Further studies are required to better understand differences between human and animal responses to SM so that more effective countermeasures can be developed that not only enhance wound healing, but also mitigate the blistering response.

## Figures and Tables

**Table 1.** Effects of sulfur mustard on guinea pig skin

Animal	Model	Citations	Strain/exposure route	Measurements/treatments	PMID #
Guinea pig	Dorsal skin	Vogt et al., 1984^19^	Guinea pig/liquid	Histopathology, TEM	6233199
		Mershon et al., 1990^15^; Braue et al., 1997^20^, 1998^17^; Snider et al, 1999^31^	Hairless guinea pig/vapor cap	Draize test, histopathology, Nikosky’s signs	2258024 27333584 27332107 10594902
		Cowan et al., 1993^94^ Cowan & Broomfield, 1993^95^	Hairless guinea pig/vapor cap	Increased proteolytic activity, inflammation	8299005 8299000
		Yourick et al., 1991^59^, 1992^96^, 1993^60^, 1995^58^	Hairless guinea pig/vapor cap	Histopathology, erythema, NAD+/NAD+/niacinamide, promethazine, indomethacin	1838996 1440603 8266337 7782559
		Petrali et al., 1993^62^, 1997^37^; Kan et al., 2003^34^	Hairless guinea pig/vapor cap	Histopathology, TEM, basement membrane, basal cell apoptosis	8462065 9144634 12696578
		Smith et al., 1995^52^, 1997^18^	Hairless guinea pig/vapor cap	Histopathology	7593821 9039976
		Kjellstrom et al., 1997^97^	Haired guinea pig, continuous flow vapor	Comparison of standard dressing vs surgical excision vs surgical excision plus autografts	9140575
		Logan et al., 1999^98^	Hairless guinea pig/vapor cap	PK/PD	10234473
		Langenberg et al., 1998^99^	Hairless guinea pig/liquid	Toxicokinetics	10028407
		Sawyer et al., 1999^100^, 2000^101^, 2008^102^; Mi et al., 2003^103^	Hairless guinea pig/vapor cap	Draize test, pathology, apoptosis, p53/hypothermia, L-NAME, dimercaptosuccinic acid	10413186 10662607 14613718 18516227
		Wormser et al., 1997^104^, 2002^28^; Brodsky et al., 2006^105^	Dunken Hartley and hairless guinea pigs/ liquid, vapor cap	Comparative study, toxicokinetics, histopathology/iodine	9049053 12242609 16252085
		Dachir et al., 2010^77^, 2012^22^, 2014^57^	Hairless guinea pig/vapor cap	TEWL, PGE2, MMP-2/9, histopathology/macrophages	20384890 23082902 24641113
		Mishra et al., 2010^106^	Hairless guinea pig/vapor cap	Immune sensitization, proliferation, cytokine expression	19887117
		Benson et al., 2011a^107^, 2011b^108^; Weber et al., 2011^71^	Hairless guinea pig/vapor cap	PK/PD, histopathology, erythema, edema, MMP-2/9, model development	21410818 21598172 21473735
		Barillo et al., 2017^53^	Hairless guinea pig/vapor cap	Skin permeation studies/wound dressings	28846576

**Table 2.** Effects of sulfur mustard on rat skin

Animal	Model	Citations	Strain/exposure route	Measurements/treatments	PMID #
Rat	Dorsal skin	Vojvodić et al., 1985^74^	Albino rats/liquid	Survival time, weight loss, pathology/sodium thiosulfate, vitamin E, heparin sulfate, dexamethasone, promethazine, atropine	4092884
		Black et al., 1992^109^	Wistar rats/vapor	SM metabolism, urine analysis/ thiodiglycol sulfoxide	1501468
		Hambrook et al., 1992^110^	Wistar rats/vapor cap	SM metabolism	1615709
		Kumar et al., 2002^111^	Wistar rats/liquid	LD50/ amifostine, DRDE-07	12269699
		Vijayaraghavan et al., 2005^112^	Wistar rats/liquid	LD50, histopathology, DNA fragmentation	15629193
		Kulkarni et al., 2006^113^	Wistar rats/liquid	DNA fragmentation, histopathology/ DRDE-07 analogs	16421877
		Karvaly et al., 2008^114^	Wistar rats/liquid	SM degradation/barrier creams and ointments	17429799
		Misik et al., 2013^115^	Wistar rats/liquid	Decontamination protection, LD50/Argos^™^, Dermogel^™^, FloraFree^™^	23078279
		Pohanka et al., 2013^55^	Wistar rats/liquid	Antioxidant depletion in liver, kidney, muscle	22947058
		Yue et al., 2014^116^, 2015^117^	Sprague-Dawley rats/liquid	Metabolism, DNA adducts, histopathology, weight loss, bone marrow micronucleus assay	24467472 25650027
		Wang et al., 2015^118^	Sprague-Dawley rats/liquid	Metabolism, DNA adducts	25955432
		Steinritz et al., 2021^119^	Wistar rats/liquid	SM creatine kinase B and DNA adducts	33635393

**Table 3.** Effects of sulfur mustard on mouse skin

Animal	Models	Citations	Strain/exposure route	Measurements/treatments	PMID #
Mouse	Dorsal skin	Vijayaraghavan et al., 1991^120^	Swiss mice/liquid	Survival, body weight, lipid peroxidation/ flavonoids, vitamin E, sodium thiosulfate	1926154
		Smith et al., 1997^18^	SKH1 hairless mice/liquid	Histopathology	9039976
		Rao et al., 1999^35^	Swiss mice/liquid	Systemic DNA damage	10614687
		Blank et al., 2000^121^	SKH1 hairless mice /vapor cap	Myeloperoxidase, inflammatory mediators	11428626
		Ricketts et al., 2000^122^	SKH1 hairless mice /vapor cap	Inflammatory mediators	11428647
		Kumar et al., 2001^123^	Swiss mice/liquid	Oxidative damage/ Trolox, quercetin, GSH	11248218
		Anderson et al., 2002^124^	CD1 neonatal mice/vapor cap	Histopathology	20597816
		Kumar et al., 2002^111^ Kulkarni et al., 2006^113^ Vijayaraghaven et al., 2005^112^	Swiss mice/liquid	LD50, histopathology, DNA fragmentation/ amifostine, DRDE-07 analogs	12269699 16421877 15629193
		Sharma et al., 2010^125^	Swiss mice/liquid	Mortality, hematology, GSH/GSSG, DNA fragmentation/amifostine, NAC, melatonin, thiosulphate, DRDE-07	20466873
		Vallet et al., 2012^126^	SKH-1 hairless mice/vapor cap	Inflammatory mediators	21939433
		Lomash et al., 2013^127^	Swiss albino mice/ liquid	Histopathology, inflammatory-reparative biomarkers	22672652
		Clery-Barraud et al., 2013^128^	SKH-1 hairless mice/vapor cap	TEWL, evaporimeter, cutometer, skin color change	22741598
		Mouret et al., 2015^38^ Sauvaigo et al., 2016^129^, Batal et al., 2013^4^, 2015^130^	SKH-1 hairless mice/liquid	TEWL, skin color change, histopathology, inflammatory mediators, DNA repair enzymes, DNA, GSH adducts	25275893 26551547 24141030 25562541
		Das et al., 2016^131^	C57BL6 mice/liquid	Lethality, wound area, body weight, hematology, bone marrow cellularity/ vitamin D	26940683
		Joseph et al., 2011^63^, 2014^64^, 2016^23^, 2018^48^	SKH-1 hairless mice/vapor cup	Wound healing, inflammatory markers/ anticholinergic prodrug	21672537 24662110 27371823 29127031

**Table 4.** Effects of sulfur mustard in the mouse ear vesicant model

Animal	Model	Citations	Strain/exposure route	Measurements/treatments	PMID #
Mouse	Mouse ear vesicant model	Casillas et al.,2000^69^, Smith et al., 1997^18^	CD1 mice/liquid	Edema, histopathology, inflammatory meditators/Olvanil, steroids, NSAIDs	11428628 9039976
		Monteiro-Riviere et al., 1999^36^	CD1 mice/liquid	Dermal edema, basement membrane proteins	10513676
		Sabourin et al., 2000^70^	CD1 mice/liquid	Inflammatory mediators	11083082
		Ricketts et al., 2000^122^	CD1 mice/liquid	Inflammatory mediators	11428647
		Powers et al., 2000^66^	CD1 mice/liquid	Serine and cysteine proteases, elastase, metalloproteases	11428632
		Dachir et al., 2004^11^	CD1 mice/liquid	Histopathology, inflammatory mediators/ steroids, NSAIDs	15052605
		Gerecke et al., 2009^132^	CD1 mice/liquid	Microarrays/MMP2, MMP9 inhibitors	18955075
		Chang et al., 2018^133^, 2020a^39^, 2020b^61^	CD1 mice/liquid	Inflammatory mediators, epidermal hyperplasia, microblisters, laminin γ2 proteolytic fragments/type IV collagenase inhibitor	29935281 32421930 32479919

**Table 5.** Effects of sulfur mustard on rabbit skin

Animal	Model	Citations	Strain/exposure route	Measurements/treatments	PMID #
Rabbit	Dorsal/ventral skin	Vogt et al., 1984^19^	Rabbit/liquid	Histopathology, TEM/ hydrocortisone	6233199
		Vojvodić et al., 1985^74^	Chinchilla rabbit/ liquid	Skin lesions pathology/ sodium thiosulfate, dexamethasone, promethazine	4092884
		Dannenberg et al.,1985^44^ Harada et al., 1985^134^, 1987^45^ Higuchi et al., 1988^135^ Tsuruta et al., 1997^136^ Tanaka et al., 1997^46^	New Zealand white rabbits/liquid	*In vivo-in vitro* studies, histopathology, release of inflammatory mediators, proteases, chemoattractant	4050973 4050975 2433944 3049342 8796382 9187966
		Chauhan et al., 1996^43^	New Zealand white rabbits /liquid	Histopathology, scanning electron microscopy, extracellular matrix	8956094
		Liu et al., 1999^73^	Rabbits/liquid	Lesion size, erythema/ topical skin protectants	10594900
		Kumar et al., 2010^137^	New Zealand white rabbits/liquid	Weight change, erythema/ amino alylaminoethane thiols	20164158
		Zhang et al., 2014^138^ Lin et al., 2014^139^ Nie et al., 2014^140^	Domestic rabbits/ liquid	SM metabolism, DNA adducts, GSH adducts	24858262 24361979 24924210
		Sun et al., 2015^75^	New Zealand rabbits/liquid	Histopathology/decontamination with potassium ketoxime	24641121
	Hind limb	Hansen et al., 1951^141^	Albino rabbits/ liquid	Appearance of lesions/ hypothermia	14923336
	Rabbit ear	Schoene et al., 1989^76^	Albino rabbits/continuous flow vapor exposure cell	Erythema, dose, permeation	2596397
		Zlotogorski et al.,1997^71^	Albino rabbits/ liquid	Draize, edema, erythema, histopathology	9184197

**Table 6.** Effects of sulfur mustard on pig skin

Animal	Model	Citations	Strain/exposure route	Measurements/treatments	PMID #
Pig/minipig	Dorsal skin models	Lindsay et al., 1995^142^	Yucatan miniature swine/vapor cap	Collagen, glycoprotein, histopathology	7598994
		Smith et al., 1996^21^	Weanling pig/liquid	Histopathology	8902098
		Brown et al., 1997^143^	Yucatan minipig/vapor cap	Histopathology, transmission electron microscopy	9166101
		Smith et al., 1997a^18^, 1997b^87^	Yorkshire cross weanling pig/vapor cap	Basement membrane proteins, proliferation, apoptosis	9039976 9302645
		Logan et al., 2000^144^	Yorkshire cross weanling pig/vapor cap	Measurement of SM skin off gassing	11428637
		Reid et al., 2000^42^	Yorkshire weanling pig/vapor cap	Clinical evaluation, histopathology	11428629
		Chilcott et al., 2000^145^, 2007^88^	White pig/vapor cap	TEWL, chromameter, skin reflectance spectroscopy, histopathology/pretreat with barrier cream	10741590 17687688
		Sabourin et al., 2002^86^	Yorkshire weanling pig/vapor cap	Proinflammatory markers	12481301
		Hall et al., 2017^50^	White pig/liquid	Scanning laser Doppler, skin reflectance spectroscopy, thermography, histopathology/WoundStat^™^	28304107
		Dachir et al., 2017^10^	White pig/vapor cap or liquid	Erythema, histology, cholinesterase inhibition/Dermostyx (IB1)	27417258
		Laskin et al., 2020^40^	Gottingen minipig/vapor cap	Histopathology, keratinocyte proliferation, growth and differentiation markers	32445752
		Barillo et al., 2017^53^, 2020^92^	Gottingen minipig/vapor cap	Skin permeation studies/wound dressings, TEWL, histopathology/ methods of debridement	28846576 31504620
		Dachir et al., 2021^146^	White pig/vapor cap	Erythema, cholinesterase inhibition/ decontamination- Fuller’s Earth, oxime lotion	33508307

**Table 7.** Effects of sulfur mustard on pig skin

Animal	Model	Citations	Strain/exposure route	Measurements/treatments	PMID #
Pig	Ventral skin models	Graham et al. 2002^49^	Yorkshire cross weanling pig/ vapor cap	Elasticity, scanning laser Doppler, TEWL, chromometer	12005121
		Graham et al., 2006^147^	Yorkshire weanling cross pig/liquid	Histopathology/laser debridement, hydrocolloid wound dressings	17111042
		Reid et al., 2000^42^, 2007^32^	Yorkshire weanling cross pig/liquid	Chromometer, TEWL, scanning laser Doppler, histopathology	11428629 17374066
		Rogers et al., 2008^148^	Yorkshire crossbred pig/liquid	Transcript analysis, porcine genome arrays	18988085
		Price et al., 2009^149^	Yorkshire crossbred pig/liquid	Transcriptional analysis, porcine genome arrays	19694609
		Graham et al., 2009^33^	Yorkshire weanling crossbred pig/vapor cap	Clinical measurements (TEWL, chromometer, torsional ballistometry, ultrasonography), histopathology, basement membrane proteins /Amino-Plex^®^, Aquacel^®^	18762227
		Plahovinsak et al., 2016^89^	Yorkshire or Yorkshire crossbred pig/ liquid	TEWL, Draize, chromometer, histopathology/clobetasol propionate, diclofenac sodium, capsaicin	26362124

**Table 8.** Skin histopathology scoring for evaluating sulfur mustard countermeasures using Göttingen minipigs^a^

Marker	Scoring
Re-epithelialization	0 - epithelium does not completely cover wound (wound is still open) 1 - wound is completely closed with epithelial cell monolayer 2 - wound is completely covered 3 - wound is completely covered by at least 2 layers of epithelial cells and includes at least the presence of some stratum corneum. 4 - wound is completely covered by greater that 2 layers of epithelial cells and includes normal corneum
Abnormal epidermal cells	dyskeratosis, apoptosis, pyknosis, karyorrhexis 0 - present (over and above control background levels) 1 - absent (as compared to control background incidence)
Basement membrane	0 - basement membrane is not completely intact 1 - basement membrane is intact with abnormal architecture 2 - basement membrane is intact with normal architecture
Hair follicles	0 - absent/abnormal as compared to control background 1 - absent/normal as compared to control background
Dermal inflammation	3 - no increase in leukocytes over that seen in normal untreated skin 2 - increased leukocytes noted in papillary dermis 1 - increased leukocytes in papillary dermis and upper half of reticular dermis 0 - diffuse increased leukocytes extending to all layers of the dermis
Rete ridges	0 - absent, 1 – present
Vascular proliferation	1 - absent, 2 – present
Hemorrhage	1 - absent, 0 – present

a Scoring system^33,92^
